# Role of Oxidative Stress and Ca^2+^ Signaling in Psychiatric Disorders

**DOI:** 10.3389/fcell.2021.615569

**Published:** 2021-02-11

**Authors:** Akito Nakao, Yoshihiro Matsunaga, Katsumi Hayashida, Nobuaki Takahashi

**Affiliations:** ^1^Laboratory of Molecular Biology, Department of Synthetic Chemistry and Biological Chemistry, Graduate School of Engineering, Kyoto University, Kyoto, Japan; ^2^The Hakubi Center for Advanced Research, Kyoto University, Kyoto, Japan

**Keywords:** oxidative stress, Ca^2+^ signaling, TRP channels, behavior, psychiatric disorders

## Abstract

Psychiatric disorders are caused by complex and diverse factors, and numerous mechanisms have been proposed for the pathogenesis of these disorders. Accumulating evidence suggests that oxidative stress is one of the general factors involved in the pathogenesis/pathophysiology of major psychiatric disorders, including bipolar disorder, depression, anxiety disorder, and schizophrenia. Indeed, some clinical trials have shown improvement of the symptoms of these disorders by antioxidant supplementation. However, the molecular basis for the relationship between oxidative stress and the pathogenesis of psychiatric disorders remains largely unknown. In general, Ca^2+^ channels play central roles in neuronal functions, including neuronal excitability, neurotransmitter release, synaptic plasticity, and gene regulation, and genes that encode Ca^2+^ channels have been found to be associated with psychiatric disorders. Notably, a class of Ca^2+^-permeable transient receptor potential (TRP) cation channels is activated by changes in cellular redox status, whereby these TRP channels can link oxidative stress to Ca^2+^ signals. Given the unique characteristic of redox-sensitive TRP channels, these channels could be a target for delineating the pathogenesis or pathophysiology of psychiatric disorders. In this review, we summarize the outcomes of clinical trials for antioxidant treatment in patients with psychiatric disorders and the current insights into the physiological/pathological significance of redox-sensitive TRP channels in the light of neural functions, including behavioral phenotypes, and discuss the potential role of TRP channels in the pathogenesis of psychiatric disorders. Investigation of redox-sensitive TRP channels may lead to the development of novel therapeutic strategies for the treatment of psychiatric disorders.

## Introduction

Psychiatric disorders, which are chronic, recurrent, and devastating disorders, are one of the main causes of disability worldwide, with the current understanding of psychiatric disorders remaining limited due to the complex and diverse nature of these disorders. Based on a 2017 survey, the number of patients with psychiatric disorders is extremely high worldwide (284 million cases of anxiety disorders; 264 million cases of depression; 46 million cases of bipolar disorder; 20 million cases of schizophrenia) (Ritchie and Roser, 2018); therefore, therapeutic strategies for the treatment of psychiatric disorders are urgently required.

Accumulating evidence suggests that oxidative stress is one of the general factors involved in the pathogenesis/pathophysiology of psychiatric disorders (Ng et al., 2008), with antioxidant levels seeming to correlate with the degree of severity of psychiatric disorders (Zhang et al., 2003; Machado-Vieira et al., 2007; Sarandol et al., 2007), and antioxidant treatment has been shown to improve psychiatric symptoms in some clinical trials (Dakhale et al., 2005; Sivrioglu et al., 2007; Ellegaard et al., 2019). Moreover, markers of oxidative stress, including lipid peroxidation products and oxidized DNA, have been shown to be elevated in the blood of patients with psychiatric disorders. While the molecular mechanisms by which oxidative stress induces psychiatric disorders are still largely unknown, the lines of evidence strongly suggest that oxidative stress is associated with the physiology/pathology of psychiatric disorders.

Ca^2+^ channels mediate a number of neuronal functions, including neuronal excitability, neurotransmitter release, synapticplasticity, and gene regulation. Many lines of evidence from human studies have indicated a critical contribution of voltage-dependent Ca^2+^ channels (VDCC) to the pathogenesis/pathophysiology of psychiatric disorders (Sklar et al., 2002, 2011; Ferreira et al., 2008; Ripke et al., 2011, 2014). However, since VDCCs control multiple critical physiological functions in the central nervous system (CNS), targeting VDCCs could cause complicated and uncontrollable results. Thus, targeting the “cart” that modulates Ca^2+^ signaling upon oxidative stress rather than the “horse” that governs the CNS system may be a promising approach and could reduce unwanted side effects.

Among the Ca^2+^-permeable cation channels, transient receptor potential (TRP) channels have attracted attention as unique sensors of a wide variety of stresses, including oxidative stress (Mori et al., 2016). Given that redox-sensitive TRP channels induce Ca^2+^ influx upon oxidative stress, these channels could be mediators that link oxidative stress to dysregulated Ca^2+^ signaling in the pathogenesis/pathophysiology of psychiatric disorders.

In the first part of this review, we introduce the literature that examines oxidative stress in psychiatric disorders. In the second part, we review the significance of Ca^2+^ signaling in psychiatric disorders and provide current insights into the roles of redox-sensitive TRP channels in the functioning of neurons and glia as well as the development of neuronal connectivity and the function of the higher brain.

## Oxidative Stress in Psychiatric Disorders

**FIGURE 1:**
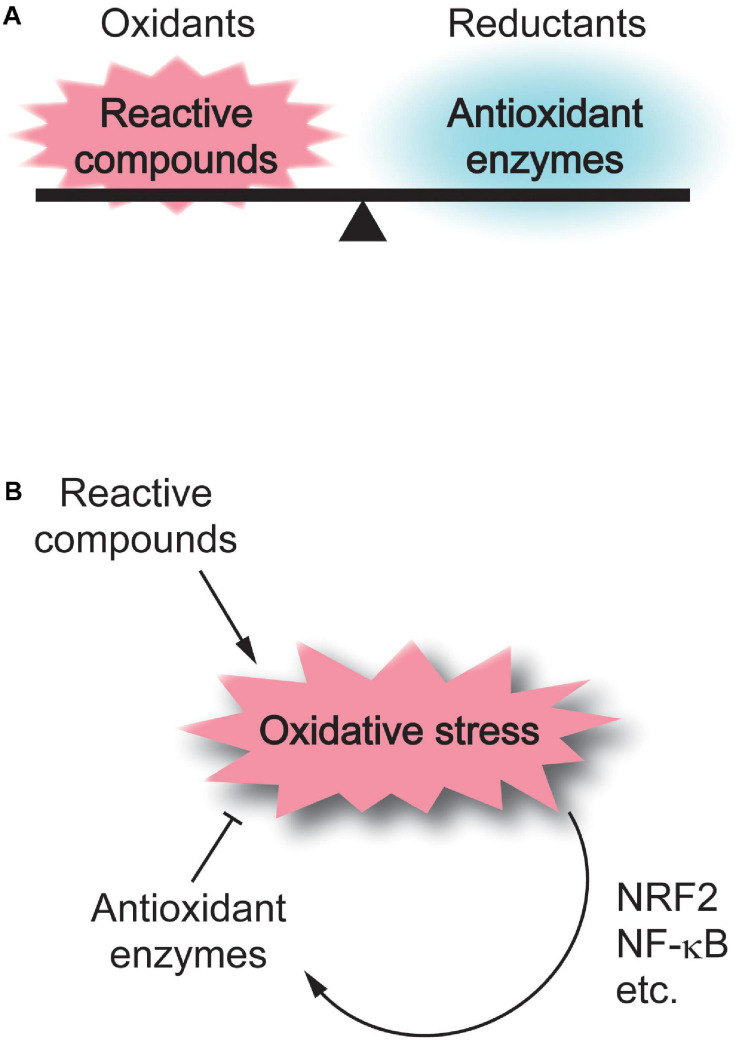
Regulation of cellular redox homeostasis. **(A)** Cellular redox status is determined by the balance between the levels of intracellular reactive compounds and antioxidant enzymes. **(B)** Reactive compounds shift the cellular redox status to oxidative conditions, while antioxidant enzymes neutralize oxidative stress. The expression of antioxidant enzymes is induced upon oxidative stress via oxidant defense transcription factors, such as NRF2 and NF-κB.

Cellular redox status is determined by the balance between the levels of intracellular antioxidants and redox reactive species, including reactive oxygen species (ROS) and other electrophilic molecules, which can cause oxidative damage to membrane lipids, proteins, and DNA (Figure 1A). While a decrease in antioxidant enzymes shifts the cellular redox status to oxidative conditions, intriguingly, the expression of antioxidant enzymes is induced upon oxidative stress via oxidant defense transcription factors, such as NRF2 and NF-κB; therefore, both enhanced and suppressed antioxidant expression can be associated with oxidative stress (Figure 1B).

It is highly controversial whether the antioxidant system is upregulated or downregulated in psychiatric disorders. In bipolar disorder, the activities of superoxide dismutases (SODs) and catalase (CAT) have been found to be decreased in the blood (Ranjekar et al., 2003), while the activity of glutathione peroxidases (GPXs) has been shown to be comparable between bipolar patients and healthy individuals (Kuloglu et al., 2002c; Ranjekar et al., 2003).

In contrast, several studies have demonstrated increased SOD activities in bipolar disorder (Abdalla et al., 1986; Kuloglu et al., 2002c). In major depression and anxiety disorders, such as obsessive-compulsive disorder, panic disorder, and social phobia, the activity of SODs alone or SODs and GPXs has been shown to be increased (Kuloglu et al., 2002a, b; Khanzode et al., 2003; Atmaca et al., 2004; Sarandol et al., 2007). In schizophrenia, higher activities of SODs (Abdalla et al., 1986; Kuloglu et al., 2002c) and GPXs have been reported in the blood of patients (Kuloglu et al., 2002c), while several other studies have reported that the activities of SODs, CAT, and GPXs are lower in schizophrenic patients than in healthy controls (Ranjekar et al., 2003; Ben Othmen et al., 2008).

Some studies have reported a correlation between antioxidant activities in the blood and the degree of severity of psychiatric disorders. In a finding which suggests that antioxidant level is an indicator of disease severity and that lithium, a primary treatment for bipolar disorder, directly or indirectly normalizes redox status, one study that compared healthy controls with either unmedicated or lithium-treated patients in manic episodes of bipolar disorder indicates that, while SOD activity was significantly higher in manic patients compared with controls, it was indistinguishable between the lithium-treated group and the control group (Machado-Vieira et al., 2007). In patients with major depressive disorder, a significant positive correlation was found between the severity of the disease and SOD activity (Sarandol et al., 2007). Interestingly, serotonin reuptake inhibitors (SSRIs), which are antidepressant drugs, have been shown to decrease SOD activity (Bilici et al., 2001; Khanzode et al., 2003); however, SSRI treatment has also been reported to increase SOD activity in patients with major depressive disorder (Herken et al., 2007). In schizophrenic patients, SOD activity is significantly increased in the serum compared to control subjects and is decreased after treatment with atypical antipsychotics, such as clozapine, risperidone, olanzapine, quetiapine, and ziprasidone, in schizophrenic patients (Dakhale et al., 2004). It has also been reported that treatment with risperidone, an atypical antipsychotic, significantly decreased the blood SOD levels in schizophrenic patients and that decreased SOD levels may correlate with an improvement in symptoms (Zhang et al., 2003).

In contrast to the controversy on the effects of antioxidant activityon psychiatric disorders, overall, markers of oxidative stress, such as lipid peroxidation and oxidized DNA, have been shown to be elevated in patients with psychiatric disorders. An increase in one lipid peroxidation product, malondialdehyde (MDA), was reported in both bipolar disorder and schizophrenia (Kuloglu et al., 2002c). The level of thiobarbituric acid-reactive substances, another marker of lipid peroxidation, has also been reported to be increased in schizophrenia (Herken et al., 2001; Akyol et al., 2002). In major depression, a number of factors that maintain or disturb redox homeostasis are altered: depletion of ω-3 fatty acids, which suppress lipid peroxidation (Peet et al., 1998), elevation of MDA (Bilici et al., 2001; Khanzode et al., 2003; Sarandol et al., 2007), and increases in 8-hydroxy-2′-deoxyguanosine, a marker of oxidized DNA damage (Forlenza and Miller, 2006). In anxiety disorders, elevated MDA levels have been reported in obsessive-compulsive disorder (Kuloglu et al., 2002a; Ersan et al., 2006), panic disorder (Kuloglu et al., 2002b), and social phobia (Atmaca et al., 2004). These results provide evidence that oxidative stress is induced in psychiatric disorders and suggest both that the antioxidant system is upregulated through oxidant defense mechanisms and that decreased antioxidant activities lead to enhanced oxidative stress in some types of psychiatric disorders (Figure 1).

The potential significance of oxidative stress in psychiatricdisorders has been demonstrated by some clinical trials that reportedan improvement in symptoms through antioxidant treatment(Table 1). In patients with bipolar disorder, a randomized, double-blind, multicenter, placebo-controlled study of individuals (*n* = 75) showed that 24-week treatment with *N*-acetyl cysteine (NAC) significantly improved depressive symptoms (Berk et al., 2008), while a three-arm, 16-week, double-blind, randomized, placebo-controlled NAC treatment trial (*n* = 181) with depressive symptoms in bipolar disorder provided overall negative results, with no significant differences between groups detected at the primary outcome but some positive secondary signals were detected (Berk et al., 2019), suggesting that longer-term supplementation of NAC is required for the amelioration. In patients with major depressive disorder, a double-blind, randomized, placebo-controlled, clinical trial was performed with NAC as an adjunctive treatment in 269 participants, and the results exhibited limited but significant effects of adjunctive NAC treatment in reducing depressive symptoms in patients with major depressive disorder (Berk et al., 2014). Notably, meta-analysis was carried out to aggregate the data on double-blind, randomized, placebo-controlled trials evaluating the effect of NAC treatment on depressive symptoms in a total of 574 participants, of whom 291 were randomized to receive NAC and 283 received a placebo, regardless of the main psychiatric conditions. The results demonstrated that treatment with NAC significantly improved depressive symptoms in patients with bipolar and major depressive disorders and in individuals with trichotillomania or heavy smoking (Fernandes et al., 2016). In patients with schizophrenia, a 12-week, double-blind, randomized, placebo-controlled, clinical trial has recently been performed to assess the effectiveness of NAC as an adjunctive treatment with conventional antipsychotic medications in 84 patients (Sepehrmanesh et al., 2018). This clinical trial demonstrated that NAC-treated patients showed significantly improved cognitive functions (Sepehrmanesh et al., 2018). In addition to NAC supplementation, co-treatment of antipsychotic drugs with both ω-3 fatty acids and vitamins E and C for 4 months improved psychotic symptoms in schizophrenic patients (Arvindakshan et al., 2003). There results suggest that oxidative stress is involved in the pathophysiology of psychiatric disorders and that antioxidant supplementation may have a suppressive effect on symptoms.

**TABLE 1:** Clinical trials for antioxidant treatment in patients with schizophrenia, bipolar disorder, and major depressive disorder.

Schizophrenia			

	Trial design		
Antioxidant	→ Outcomes	Sample size	
**NAC** (Sepehrmanesh et al., 2018)	Adjunctive NAC 1,200 mg/day supplements	NAC:	42
	12 weeks double-blind treatment	Placebo:	42
	→ Improvement in PANSS, MMSE, Digit Span Test, andSCWT scores		
**Vitamin C/E and ω-3 fatty acids** (Sivrioglu et al., 2007)	Adjunctive vitamin C 1,000 mg/day, vitamin E 800 IU/day, EPA 360 mg/day, and DHA 240 mg/day supplements	Vitamin C/E and ω-3 fatty acids:	17
	4 months open-label treatment		
	→ Improvement in BPRS, BARS, SAS scores, and SANS total score		
**Vitamin C** (Dakhale et al., 2005)	Adjunctive vitamin C 500 mg/day supplements	Vitamin C:	20
	8 weeks double-blind treatment	Placebo:	20
	→ Improvement in BPRS score		
**Vitamin C/E and ω-3 fatty acids** (Arvindakshan et al., 2003)	Adjunctive vitamin C 1,000 mg/day, vitamin E 800 IU/day, EPA 360 mg/day, and DHA 240 mg/day supplements	Vitamin C/E and ω-3 fatty acids:	33
	4 months open-labeled treatment and 4 months follow up		
	→ 8 months; Improvement in BPRS-total, positive-PANSS, general psychopathology-PANSS, and PANSS-total scores		
**Bipolar disorder**			

	**Trial design**		
**Antioxidant**	**→ Outcomes**	**Sample size**	

**NAC** (Berk et al., 2019)	Adjunctive NAC 2,000 mg/day and cocktail of 16 nutrient agents supplements	NAC + nutrient agents:	61
	16 weeks double-blind treatment and 4 weeks follow up	NAC:	59
	→ 16 weeks; No significant change	Placebo:	61
	20 weeks; Improvement in MADRS, BDRS, SOFAS, and LIFE-RIFT scores (Placebo v.s. NAC + nutrient agents)		
**NAC** (Ellegaard et al., 2019)	Adjunctive NAC 3,000 mg/daysupplements	NAC:	40
	20 weeks double-blind treatment and 4 weeks follow up	Placebo:	40
	→ 20 weeks; Improvement in YMRS score		
**NAC** (Berk et al., 2008)	Adjunctive NAC 2000 mg/daysupplements	NAC:	38
	24 weeks double-blind treatment and 4 weeks follow up	Placebo:	37
	→ 24 weeks; Improvement in MADRS, BDRS, CGI-S-BP, Q-LES-Q, LIFE-RIFT, SLICE-LIFE, GAF, and SOFAS scores		
**Major depressive disorder**			

	**Trial design**		
**Antioxidant**	**→ Outcomes**	**Sample size**	

**NAC** (Berk et al., 2014)	Adjunctive NAC 2,000 mg/daysupplements	NAC:	135
	12 weeks double-blind treatment and 4 weeks follow up	Placebo:	134
	→ 16 weeks; Improvement in MADRS, CGI-S, SLICE-LIFE, and LIFE-RIFT scores		

**BARS, Barnes Akathisia Rating Scale; BDRS, Bipolar Depression Rating Scale; BPRS, Brief Psychiatric RatingScale; CGI-S, Clinical Global Impressions-Severity of illness; CGI-S-BP, Clinical Global Impression Severity scale for Bipolardisorder; GAF, Global Assessment of Functioning Scale; LIFE-RIFT, Longitudinal Interval Follow-Up Evaluation-Range of ImpairedFunctioning Tool; MADRS, Montgomery-Åsberg Depression Rating Scale; MMSE, Mini-Mental State Examination; PANSS, Positive And Negative Syndrome Scale; Q-LES-Q, Quality of Life Enjoyment and Satisfaction Questionnaire; SANS, Scale for the Assessment of Negative Symptoms; SAS, Simpson-Angus Scale; SCWT, Stroop Color and Word test; SLICE-LIFE, Streamlined Longitudinal Interview Clinical Evaluation from the Longitudinal Interval Follow-up Evaluation; SOFAS, Social and Occupational Functioning Assessment Scale; YMRS, Young Mania Rating Scale.**

## Ca^2+^ Signaling in Psychiatric Disorders

In the CNS, Ca^2+^ signaling is pivotal for numerous cellular events, including neuronal excitability, neurotransmitter release, synaptic plasticity, and Ca^2+^-induced gene regulation (Catterall, 2011; Sawamura et al., 2017). Due to the fundamental role of Ca^2+^ signaling in the CNS, Ca^2+^ channels are thought to be a prime target for the pathogenesis of psychiatric disorders. Indeed, genes encoding Ca^2+^ channels have been shown to be associated with psychiatric disorders (McQuillin et al., 2006; Ripke et al., 2014; Griesi-Oliveira et al., 2015).

Among the Ca^2+^ channels, *CACNA1C*-encoded Ca_V_1.2, an L-type VDCC, is strongly associated with psychiatric disorders (Sklar et al., 2002, 2011; Ferreira et al., 2008; Ripke et al., 2011, 2014). In humans, a risk-associated single nucleotide polymorphism (rs1006737) in *CACNA1C* predicts both increased hippocampal activity during emotional processing and higher prefrontal activity during executive cognition (Bigos et al., 2010). In rodents, conditional deletion of *Cacna1c* in the hippocampus and cortex results in severe impairment of hippocampus-dependent spatial memory based on the Morris water maze test (Moosmang et al., 2005; White et al., 2008). Acute pharmacological blockade of Ca_V_1.2, but not chronic genetic inactivation, impairs the acquisition of fear learning (Langwieser et al., 2010), while anterior cingulate cortex-limited deletion of *Cacna1c* in mice impairs observational fear learning (Jeon et al., 2010). Constitutive *Cacna1c* heterozygous knockout (KO) mice, forebrain-specific conditional *Cacna1c* KO mice, and prefrontal cortex-specific *Cacna1c* knockdown mice show increased anxiety-like behavior in the elevated-plus maze test (Lee et al., 2012). The behavioral phenotypes in mice with genetic or pharmacological inhibition of Ca_V_1.2 are summarized in the previous literature (Nakao et al., 2015).

Clinical studies with Ca_V_1.2 blockers for psychiatric disorders have been summarized in a previous review paper (Kabir et al., 2017). While some clinical trials using Ca_V_1.2 blockers have demonstrated improved symptoms of psychiatric disorders, the effect remains controversial (Kabir et al., 2017). Notably, since Ca_V_1.2 controls multiple critical physiological functions in various organs and tissues, including the vascular system, the sinoatrial node, cardiomyocytes, pancreatic islets, adrenal medulla, and intestinal smooth muscle (Zamponi et al., 2015), the use of Ca_V_1.2 blockers could cause highly complicated and uncontrollable results.

## Redox-Sensitive TRP Channels

Among the Ca^2+^-permeable channels, a class of TRP channels is activated by oxidative stress (so-called redox-sensitive TRP channels). Given the critical role of oxidative stress and aberrant Ca^2+^ signaling in psychiatry disorders, it is possible that redox-sensitive TRP channels serve as a cue for understanding the molecular pathogenesis of these disorders.

The *trp* was originally identified through the genetic studies of the *Drosophila* phototransduction mutant (Montell and Rubin, 1989). The term “TRP” is derived from “transient receptor potential” as the *trp* mutant photoreceptors fail to generate the Ca^2+^-dependent sustained phase of receptor potential and therefore fail to induce subsequent Ca^2+^-dependent adaptation to light in *Drosophila*. In mammals, 28 TRP homologs have been identified since the cloning of the *Drosophila trp* gene (Montell and Rubin, 1989). They are divided into six subfamilies: TRPC (canonical), TRPV (vanilloid), TRPM (melastatin), TRPP (polycystin), TRPML (mucolipin), and TRPA (ankyrin) groups, according to the amino acid homology (Clapham, 2003; Nilius et al., 2007). TRP channels are putative six-transmembrane polypeptide subunits and assemble as tetramers to form a variety of Ca^2+^-permeable cation channels. Because of their distinct activation mechanisms and biophysical properties, TRP channels are highly suited to function in sensory receptor cells, either as molecular sensors for environmental or endogenous stimuli or as modulators of signal transduction cascades downstream of metabotropic receptors. Indeed, TRP channels play crucial roles in many types of senses, including touch, taste, and smell in mammals (Clapham, 2003; Voets et al., 2005; Moran et al., 2011). Thus, in marked contrast to VDCCs and fast ligand-gated Ca^2+^-permeable channels that participate in specialized cellular functions, TRP channels play divergent roles in cell physiology and pathophysiology.

Among TRP channels, one particular group of TRP channels, including TRPM2, TRPC5, TRPV1–V4, and TRPA1 channels, function as exquisite sensors of redox status. The redox-sensitive TRP channels induce Ca^2+^ entry into the cells in response to cellular redox perturbation and can convert oxidative stress information into Ca^2+^ signals. Details of redox-sensitive TRP channels have been summarized in several review papers from our group (Takahashi and Mori, 2011; Takahashi et al., 2011a; Kozai et al., 2013; Sakaguchi and Mori, 2020).

TRPM2 channel, the first redox-sensitive TRP channel to be identified, is activated by H_2_O_2_ through the production of nicotinamide adenine dinucleotide and its metabolites, ADP-ribose and cyclic ADP-ribose (Hara et al., 2002; Perraud et al., 2005). A later study proposed that oxidation of the methionine located in the ADP-ribose binding region of TRPM2 sensitizes the channel activities (Kashio et al., 2012). H_2_O_2_-activated Ca^2+^ or cation influx through TRPM2 induces cell death (Hara et al., 2002) and insulin secretion in pancreatic β-cells (Togashi et al., 2006; Uchida et al., 2011). Furthermore, studies using *Trpm2* KO mice have shown that TRPM2 widely contributes to the innate immune system in monocytes, macrophages, neutrophils, and natural killer cells (Yamamoto et al., 2008; Knowles et al., 2011; Di et al., 2012; Hiroi et al., 2013; Rah et al., 2015).

In addition to the indirect redox-sensing mechanism via TRPM2, direct redox-sensing mechanisms involving cysteine (Cys) modification have emerged as mechanisms that underly the activation of various TRP channels (Yoshida et al., 2006; Takahashi and Mori, 2011; Takahashi et al., 2011b). TRPC5 channel, for example, is activated by nitric oxide (NO), reactive disulfides, and H_2_O_2_ via the oxidative modification of Cys residues (Cys553 and Cys558) located on the N-terminal side of the pore-forming region between S5 and S6 transmembrane helices in mouse TRPC5 (Yoshida et al., 2006). Interestingly, TRPC5 is also activated by the reducing agent dithiothreitol and extracellular-reduced thioredoxin (Xu et al., 2008). Both thermosensor channels (TRPV1, TRPV3, and TRPV4) and the closest relatives of TRPC5 (TRPC1 and TRPC4) have Cys residues corresponding to Cys553 and Cys558 on the TRPC5 protein and are activated by NO, reactive disulfides, and H_2_O_2_ (Yoshida et al., 2006). The mustard oil or wasabi sensor TRPA1 channel, which is predominantly expressed in sensory and vagal neurons, is also activated via modification of Cys residues located in the N-terminal ankyrin repeat domain (Hinman et al., 2006; Macpherson et al., 2007; Takahashi et al., 2008) and has the highest oxidation sensitivity among all TRP channel family members (Takahashi et al., 2011b).

Notably, all the redox-sensitive TRP channels are expressed to some extent in the CNS and affect neuronal and glial functions. Given this fact, together with the evidence that both Ca^2+^ signals and oxidative stress are critical mediators in psychiatric disorders, it is possible that redox-sensitive TRP channels could be a target for delineating the pathogenesis or pathophysiology of psychiatric disorders.

## Neuronal and Glial Functions of Redox-Sensitive TRP Channels

In neuronal circuits, excitation-inhibition balance is considered a framework for understanding the mechanisms of psychiatric disorders (Sohal and Rubenstein, 2019). TRPM2 channel is abundantly expressed in the brain (Hara et al., 2002; Kraft et al., 2004; Fonfria et al., 2006) and H_2_O_2_-induced Ca^2+^ influx via TRPM2 in neurons was initially identified to induce cell death (Kaneko et al., 2006). Later studies have suggested several important roles for TRPM2 in the physiology of the CNS. TRPM2 activation by ADPR has been suggested as a regulator of VDCC and NMDAR via a rise in intracellular Ca^2+^ concentration ([Ca^2+^]_i_) in cultured hippocampal pyramidal neurons (Olah et al., 2009). In CA3-CA1 synapses, the loss of TRPM2 selectively impairs NMDAR-dependent long-term depression (LTD), while it does not affect long-term potentiation (LTP) (Xie et al., 2011). The impaired LTD in *Trpm2* KO mice is rescued by dopamine D2 receptor stimulation through the reduction of phosphorylated GSK-3β, suggesting that TRPM2 plays a key role in hippocampal synaptic plasticity via the GSK-3β signaling pathway. In guinea pig midbrain slices, TRPM2 has been shown to be required for NMDA-induced burst firing and to contribute to H_2_O_2_-dependent modulation of substantia nigra pars reticulata (SNr) GABAergic neurons (Lee et al., 2013). Given that an increase in burst firing in SNr GABAergic neurons is observed in Parkinson’s disease and that oxidative stress is linked to Parkinson’s disease, TRPM2 could be associated with the pathophysiology of Parkinson’s disease.

Quantitative post-mortem investigations have convincingly demonstrated abnormalities in the glial cells of patients with psychiatric disorders (Cotter et al., 2001). In addition to neurons and immune cells, TRPM2 has been shown to be functionally expressed in glial cells, such as the microglia and astrocytes (Kraft et al., 2004; Kaneko et al., 2006; Bond and Greenfield, 2007). Oxidative stress induced by the inhibition of intracellular GSH biosynthesis with D,L-buthionine-*S,R*-sulfoximine (BSO) elicits Ca^2+^ influx potentially via TRPM2 in astrocytes isolated from humans (Lee et al., 2010). In rat cultured astrocytes, *tert*-butyl hydroperoxide-induced oxidative stress upregulates TRPM2 mRNA within 1 h – the transcripts were peaked at 4 h and were still apparent at 24 h post-stress (Bond and Greenfield, 2007), suggesting that glial TRPM2 expression is enhanced upon oxidative stress in disease conditions, including psychiatric disorders, and mediates the pathophysiology of disease.

TRPC5 is predominantly expressed in the brain, where it can formheterotetrameric complexes with TRPC1 and TRPC4 channel subunits. Recently, TRPC1, TRPC4, and TRPC5 have been demonstrated to assemble into heteromultimers in mouse brains, but not with other TRP family members, based on quantitative high-resolution mass spectrometry (Bröker-Lai et al., 2017). Brain slices prepared from *Trpc5* KO and *Trpc4* KO mice exhibit significant reductions in the synaptic responses that are mediated by the activation of G_q/__11_ protein-coupled receptors (e.g., group I metabotropic glutamate receptors and cholecystokinin [CCK] 2 receptors) in the amygdala (Riccio et al., 2009, 2014). As a consequence, deficiency of *Trpc5* or *Trpc4* induces anxiolytic behavior in mice (Riccio et al., 2009, 2014) (see “Redox-Sensitive TRP Channels in Higher Brain Functions” section). In hippocampal neurons from *Trpc1*/*Trpc4*/*Trpc5* triple KO mice, action potential-triggered excitatory postsynaptic currents (EPSCs) are significantly reduced, whereas the frequency, amplitude, and kinetics of quantal miniature EPSC signaling remains unchanged (Bröker-Lai et al., 2017). Consistent with this observation, evoked postsynaptic responses in hippocampal slice recordings and transient potentiation after tetanic stimulation have been reported to be decreased in triple KO mice (Bröker-Lai et al., 2017). In layer III pyramidal neurons in the entorhinal cortex, enhancement of neuronal excitability induced by CCK, one of the most abundant neuropeptides in the brain, is significantly inhibited by intracellular application of the antibody to TRPC5, suggesting the involvement of TRPC5 channels in neuropeptide-mediated neuronal excitability (Wang et al., 2011). TRPC5 has been also demonstrated to mediate muscarinic receptor-induced slowing after depolarization, which is observed in the pyramidal cells of the cerebral cortex (Yan et al., 2009). Interestingly, TRPC1, TRPC4, and TRPC5 channels are activated by leptin, a hormone that plays a key role in appetite, overeating, and obesity, via the Jak2-PI3 kinase-PLCγ1 pathway in hypothalamic proopiomelanocortin neurons (Qiu et al., 2010). Taken together, these lines of evidence suggest that TRPC1, TRPC4, and TRPC5 channels are involved in various physiological processes, including memory and appetite, by regulating neuronal functions.

TRPC1, TRPC4, and TRPC5 channels have also been shown to be expressed in astrocytes (Song et al., 2005; Malarkey et al., 2008). In the results suggesting that the TRPC1, TRPC4, and TRPC5 channels are functionally expressed in astrocytes and also modulate neuronal circuits, high-resolution imaging revealed that store-operated Ca^2+^ entry (SOCE) mediated by TRPC1 is linked not only functionally but also structurally to the ER Ca^2+^ stores in mouse astrocytes (Golovina, 2005). TRPC1-mediated Ca^2+^ entry in response to mechanical and pharmacological stimulation has been shown to contribute to glutamate release from rat astrocytes (Malarkey et al., 2008).

TRPV1 is broadly expressed in the brain regions, including theolfactory nuclei, cerebral cortex, dentate gyrus, thalamus, hypothalamus, periaqueductal gray, superior colliculus, locus coeruleus, and cerebellar cortex (Roberts et al., 2004). TRPV1 is involved in various forms of synaptic plasticity, which have been well-summarized in previous review papers (Kauer and Gibson, 2009; Ramírez-Barrantes et al., 2016; Sawamura et al., 2017). In the hippocampus, TRPV1 mediates LTD at excitatory synapses onto CA1 interneurons (Gibson et al., 2008). The excitatory synapses of the hippocampal CA1 interneurons are depressed by TRPV1 agonist capsaicin and the potent endogenous TRPV1 activator 12-(S)-HPETE (Gibson et al., 2008). TRPV1 activation by capsaicin or by endocannabinoid anandamide depressed somatic but not dendritic inhibitory inputs onto dentate granule cells, suggesting that TRPV1 modulates GABAergic synaptic transmission in a compartment-specific manner (Chávez et al., 2014). Interestingly, acute inescapable stress on an elevated platform suppressed LTP and facilitated LTD at the hippocampal CA3-CA1 synapse, but the TRPV1 agonist capsaicin effectively prevented these effects, suggesting that TRPV1 contributes to stress-induced impairment of spatial memory (Li et al., 2008) (see “Redox-Sensitive TRP Channels in Higher Brain Functions” section).

TRPA1, the channel that has the highest oxidation sensitivity among the TRP channels, is expressed in the brain (Stokes et al., 2006), including in the hippocampus (Lee et al., 2016), the dentate gyrus (Koch et al., 2011), the supraoptic nucleus (Yokoyama et al., 2011), the nucleus tractus solitarii (Sun et al., 2009), and the somatosensory cortex (Kheradpezhouh et al., 2017). TRPA1 is also expressed in astrocytes and regulates basal [Ca^2+^]_*i*_ (Shigetomi et al., 2012, 2013; Takizawa et al., 2018). TRPA1 inhibition, which decreases resting [Ca^2+^]_*i*_ in astrocytes, impairs interneuron inhibitory synapse efficacy by reducing GAT-3-mediated GABA transport, resulting in the elevation of extracellular GABA (Shigetomi et al., 2012). Furthermore, astrocyte TRPA1 channels are required for constitutive D-serine release into the extracellular space, which contributes to NMDA receptor-dependent LTP in CA3-CA1 synapses (Shigetomi et al., 2013). In rat astrocyte cell line C6 cells and primary cultured astrocytes, a TRPA1-mediated spontaneous rise of [Ca^2+^]_*i*_ modulated the spontaneous release of peptide hormones from astrocytes (Takizawa et al., 2018). Recently, our group reported that TRPA1 acts as an acute hypoxia sensor in brainstem astrocytes through O_2_-dependent protein translocation and triggers ATP release from astrocytes, which potentiates respiratory center activity (Uchiyama et al., 2020). Interestingly, vascular endothelial cell-specific *Trpa1* KO exacerbates cerebral infarcts following permanent middle cerebral artery occlusion in mice (Pires and Earley, 2018), suggesting that TRPA1 in cerebral artery endothelial cells is activated by oxidative stress under ischemia and protects from cerebral ischemia through the secretion of vasodilators, including NO. Taken together, TRPA1 is expressed in diverse cell types within the brain, including glial and endothelial cells, and promotes the secretion of factors that modulate neuronal circuits and vascular tonus upon oxidative stress in disease conditions.

## Redox-Sensitive TRP Channels in the Establishment of Neuronal Connectivity

Abnormalities in neurites are considered to be involved in the pathogenesis of psychiatric disorders because they can cause impaired neuronal connectivity (Penzes et al., 2011). TRPC1 positively regulates netrin-1-induced growth-cone turning in cultured *Xenopus* spinal neurons (Wang and Poo, 2005; Shim et al., 2009), while it is not involved in brain-derived neurotrophic factor (BDNF)-induced growth-cone turning in cultured rat cerebellar granule cells (Li et al., 2005). Both TRPC1 and TRPC3 channels are required for leptin-sensitive current and leptin-induced spine formation in cultured hippocampal neurons (Dhar et al., 2014). Interestingly, SOCE mediated by TRPC1 is required for the proliferation of adult hippocampal neural progenitor cells (Li et al., 2012). Activation of TRPC4 induces inhibition and arborization of neurite growth in cultured hippocampal neurons (Jeon et al., 2013). TRPC5 that interacts with the growth-cone-enriched protein stathmin 2 has been shown to be a negative regulator of neurite extension and growth-cone morphology (Greka et al., 2003). In semaphorin 3A-induced neuronal growth-cone collapse, calpain has been demonstrated to cleave and activate TRPC5 channels, suggesting that TRPC5 acts downstream of the semaphorin signaling that causes changes in neuronal growth-cone morphology (Kaczmarek et al., 2012). Neurotrophin-3-induced Ca^2+^ influx via TRPC5 inhibits neuronal dendritic growth through activation of Ca^2+^/calmodulin kinase (CaMK) IIα (He et al., 2012). TRPC5 channels have also been shown to induce the activation of Ca^2+^/calmodulin kinase kinase (CaMKK) and the γ-isoform of CaMKI (CaMKIγ), followed by the promotion of axon formation in cultured hippocampal neurons (Davare et al., 2009). In the cerebellar cortex, *Trpc5* deficiency causes the formation of long and highly branched granule neuron dendrites with impaired dendritic claw differentiation, resulting in gait and motor coordination deficits (Puram et al., 2011). Mechanistically, TRPC5 forms a complex specifically with CaMKIIβ and induces the CaMKIIβ-dependent phosphorylation of ubiquitin ligase Cdc20-APC at the centrosome, suggesting the significance of the TRPC5/CaMKIIβ/Cdc20-APC signaling axis in the regulation of dendritic morphology (Puram et al., 2011). Thus, the TRPC1, TRPC4, and TRPC5 channels are key regulators for proper establishment of neuronal connectivity.

In cultured cortical neurons, pharmacological perturbation of TRPM2 markedly increases the axonal growth, and the neurons isolated from *Trpm2* KO mice have significantly longer neurites and a greater number of spines than those isolated from control mice (Jang et al., 2014). In addition, the blockage of TRPM2 reverses lysophosphatidic acid induced suppression of neurite growth (Jang et al., 2014), suggesting that TRPM2 is a negative regulator of neurite outgrowth.

## Redox-Sensitive TRP Channels in Higher Brain Functions

Genome scanning for 47 bipolar disorder families has shown that thereis a susceptibility locus near the telomere on chromosome 21q (Straub et al., 1994). Fine mapping linkage analysis identified that *TRPM2* on chromosome 21q is associated with bipolar disorder (McQuillin et al., 2006). Two SNPs of *TRPM2* were found in the analysis: a base pair change in exon 11 of *TRPM2*, which causes an amino acid substitution (aspartic acid to glutamic acid: D543E), and a non-conservative change from arginine to cysteine (R755C). Notably, the D543E mutation induces loss of function of TRPM2 activities, and *Trpm2* KO mice exhibit bipolar disorder-related behaviors, such as mood disturbances and impairments in social cognition (Jang et al., 2015) (Table 2). Interestingly, disruption of *Trpm2* increases phosphorylation of GSK-3, which is implicated as one of the main targets of lithium (Xie et al., 2011; Jang et al., 2015). Although the significance of GSK-3 in the pathology of bipolar disorder is still debated (Alda, 2015), these observations suggest that dysregulation of GSK-3 could, at least in part, account for the bipolar disorder-related behaviors induced by hypoactive TRPM2 mutations. Furthermore, a SNP of *TRPM2* has recently been identified as a risk factor for bipolar disorder in a Japanese population (Mahmuda et al., 2020). These lines of evidence suggest a link between defects in TRPM2 activities and the pathology of bipolar disorder.

**TABLE 2:** Behavioral phenotypes in mice with genetic or pharmacological inactivation of redox-sensitive TRP channels.

	Anxiety	Memory	Other behavioral phenotype
**TRPM2 KO mice** (Jang et al., 2015)	**Increased anxiety-like behavior** Decreased time spent in open arms in EP Increased latency to Light part in LD	N/D	Decreased interaction time in social interaction test

**TRPM2 KO mice** (Andoh et al., 2019)	Normal in LD and EP Normal in social interaction test	Normal in radial maze and Y-maze tests Decreased flexibility in Barnes maze test	Normal in rotarod, forced swim, tail suspension, and prepulse inhibition tests Increased resiliency to repeated social defeat stress

**Middle-aged TRPM2 KO mice** (Kakae et al., 2019) Middle-aged: 12–16 months old Aged: 20–24 months old	N/D	**Enhanced spatial working memory** Middle-aged TRPM2 KO mice show increased alternation behavior compared with middle-aged WT in Y-maze test. **Enhanced spatial reference memory** Middle-aged TRPM2 KO mice show increased exploratory preference compared with middle-aged WT in novel object recognition test. Aged TRPM2 KO mice show increased exploratory preference compared with aged WT in novel location recognition test.	N/D

**Stressed TRPM2 KO mice** (Ko et al., 2019) **Stressed**	**Decreased anxiety-like behavior** Stressed TRPM2 KO mice show more sucrose consumption compared with stressed WT in sucrose consumption test. Stressed TRPM2 KO mice show less escaped latency compared with stressed WT in learned helplessness test. Stressed TRPM2 KO mice show less latency to feed compared with stressed WT in novelty suppressed feeding test. Stressed TRPM2 KO mice show less immobility time compared with stressed WT in forced swim test.	N/D	N/D
**Normal**	**Decreased anxiety-like behavior** Non-stressed TRPM2 KO mice show less latency to feed compared with non-stressed WT in novelty suppressed feeding test. Non-stressed TRPM2 KO mice show less immobility time compared with non-stressed WT in forced swim test.	N/D	N/D

**TRPC5 KO mice** (Riccio et al., 2009)	**Decreasedanxiety-like behavior** Increased entries in open arms in EP in center region in OF (first 5 min) Increased number of nose contacts in social interaction test	Normal in fear conditioning test	Decrease tendencyin acoustic startle response Normal in novelty-suppressedfeeding test Normal in locomotor activity in OF

**TRPC4 KO mice** (Riccio et al., 2014)	**Decreased anxiety-like behavior** Increased entry and time spent in open arms in EP in center region in OF	Normal in auditory and contextual fear conditioning tests	Normal in acoustic startle, tail suspension, and beam walking tests Normal in gait analysis Normal in total distance in OF (red light) Increased total distance in OF

**TRPC1 KO mice** (Lepannetier et al., 2018)	Normal in OF and EP	**Impaired fear memory** Decreased freezing responsein contextual and trace fear conditioning tests **Impaired spatial working memory** Decreased alternance in Y-maze tests Decreased correct arm entry in spatial novelty preference test (modified Y-maze) Normal in Morris water maze and radial maze tests	Increased distance traveled in OF in total 2 days

**TRPC1,4,5 KO mice** (Bröker-Lai et al., 2017)	N/D	**Impaired spatial working memory** Increased number of errors in T-maze and radial maze tests Normal spatial reference memory in Morris water maze test	N/D

**TRPC4/TRPC5 channel inhibitor (M084)** (Yang et al., 2015)	**M084 decreased anxiety-like behavior** Increased numberof entries in light part in the LD Increased number ofentries and time spent in open arms in EP	N/D	Normal inlocomotor activity (no difference among control, CUS, andCUS + M084) M084 rescues the immobility which is increased by CUS inforced swim test M084 rescues the latency to feed which is increased by CUS in novelty suppressed feeding test

**TRPC4/TRPC5 channel inhibitor (HC-070)** (Just et al., 2018)	**HC-070 decreased anxiety-like behavior** HC-070 rescuesopen arm entries decreased by CCK-4 in EP Decreased number of buried marble in marble burying test	Rescued the freezing time which is increased by CSD in auditory and contextual freezing tests (HC-070 inhibited formation of trauma.)	Decreased immobility time in tail suspension and forced swim tests Normal locomotor activity in OF

**TRPV1 KO mice** (Marsch et al., 2007)	**Decreased anxiety-like behavior** Increased total distance moved in light part in LD Increased time and entries in open arms in EP Normal in OF	**Impaired fear memory** Decreased freezing response in auditory and contextual fear conditioning tests	Normal in auditory response

**TRPA1 KO mice** (Lee et al., 2017)	**Decreased anxiety-like behavior** Increased time spent in open arms in EP	**Enhanced fear memory** Increased freezing in auditory fear conditioning test **Enhanced spatial reference memory** Increased cross numbers in probe trial in Morris water maze test Increased discrimination index in novel location recognition	Enhanced social recognition behavior

**Aged TRPA1 KO mice** (Borbély et al., 2019) Young: 3–4 months old. Old: 18 months old	**Decreasedanxiety-like behavior** Young TRPA1 KO mice show increased number of entries in center region compared with young WT in OF.	**Enhanced reference memory** Old TRPA1 KO mice show higher discrimination index compared with old WT in novel object recognition test. **Enhanced spatial working memory** Young TRPA1 KO mice show less reference memory error compared with young WT in radial maze test. Old TRPA1 KO mice show less exploration time compared with WT in radial maze test.	Young TRPA1 KO mice show higher velocity compared with young WT in radial maze test.

**CSD, chronic social defeat; CUS, chronic unpredictable stress; EP, elevated plus-maze test; LD, light-dark transition test; N/D, not determined; OF, open field test**

In addition to the connection between TRPM2 and bipolar disorder, accumulating evidence has indicated the impact of TRPM2 on major depressive disorder. In a mouse model of depression via chronic unpredictable stress, disruption of *Trpm2* produced antidepressant-like behaviors accompanied by reduced ROS, suppressed ROS-induced calpain activation, and enhanced phosphorylation of two Cdk5 targets, synapsin 1 and histone deacetylase 5, which are linked to synaptic function and gene expression, respectively (Ko et al., 2019). Notably, while middle-aged (12–16 months) and aged (20–24 months) wild-type (WT) mice exhibited memory dysfunction compared with young (2–3 months) WT mice, these characteristics were undetectable in *Trpm2* KO mice (Kakae et al., 2019) (Table 2), potentially owing to defects in the sensing of oxidative stress, whose levels are increased by aging (Tan et al., 2018). *Trpm2* deficiency also attenuates social avoidance induced by repeated social defeat stress in mice (Andoh et al., 2019). Under physiological conditions, a comprehensive behavioral test battery has shown that *Trpm2* KO mice exhibit no behavioral phenotypes in light/dark transition, rotarod, elevated plus maze, social interaction, prepulse inhibition, Y-maze, forced swim, cued and contextual fear conditioning, and tail suspension tests (Andoh et al., 2019), whereas *Trpm2* KO mice were also reported to exhibit bipolar disorder-related behaviors (Jang et al., 2015) (Table 2). Differences in the genetic backgrounds of mice or the animal facilities and equipment used in the behavioral analyses could explain the inconsistency of the results. Collectively, these findings suggest that disruption of *Trpm2* potentially induces behavioral phenotypes in pathological conditions that could be related to oxidative stress.

TRPC4 and TRPC5 are highly expressed in the cortex and amygdala, the regions thought to be crucial in regulating anxiety (Riccio et al., 2009, 2014). Importantly, previous works with transgenic mice suggest that TRPC4 and TRPC5 play important roles in amygdala function, and thus inhibition of TRPC4 and TRPC5 have anxiolytic effects (Riccio et al., 2009, 2014). This implicates the significance of both TRPC4 and TRPC5 in innate fear function, which represents a key response to environmental stress. *Trpc5* KO mice exhibited diminished innate fear levels in response to innately aversive stimuli (Riccio et al., 2009) (Table 2). *Trpc4* KO mice also showed suppressed G_α q/11_-dependent responses in the amygdala, which regulate fear-related behavioral processes, and decreased anxiety-like behaviors (Riccio et al., 2014) (see “Neuronal and Glial Functions of Redox-Sensitive TRP Channels” section) (Table 2). *Trpc4* knockdown in the lateral amygdala via lentivirus-mediated gene delivery of RNAi mirrored the behavioral phenotype of constitutive *Trpc4* KO mice. Given that the animal model for the analysis of innate fear responses is limited, *Trpc4* and *Trpc5* KO mice may be useful animal models for investigating the mechanisms underlying the regulation of innate fear responses. It is worth noting that there is a caveat regarding interpretation of the behavioral data because *Trpc5* KO mice have deficits in gait and motor coordination (Puram et al., 2011).

In contrast to *Trpc4* and *Trpc5* KO mice, deletion of the *Trpc1* gene impairs fear memory formation but not innate fear behavior (Lepannetier et al., 2018), suggesting that TRPC1 regulates fear-related behavioral processes through distinct mechanisms from the TRPC5- and TRPC4-mediated pathways. In hippocampal functions, deletion of *Trpc1* impaired functioning in the Y-maze task, and the triple deficiency of *Trpc1*/*Trpc4*/*Trpc5* induced reduced performance in the T-maze and radial maze tests, while both *Trpc1* single KO and *Trpc1*/*Trpc4*/*Trpc5* triple KO mice showed normal acquisition in the Morris water maze task (Bröker-Lai et al., 2017; Lepannetier et al., 2018), suggesting that TRPC1 and possibly both TRPC4 and TRPC5 regulate spatial working memory but not spatial reference memory.

Pharmacological analysis in rats revealed that the TRPV1 antagonist, capsazepine, attenuates anxiety-like behaviors in the elevated-plus maze test (Kasckow et al., 2004). Consistent with this observation, *Trpv1* KO mice exhibited less anxiety-related behaviors in the elevated-plus maze test and in the light/dark test compared to their WT littermates (Marsch et al., 2007) (Table 2). Furthermore, *Trpv1* KO mice showed less freezing in auditory and contextual fear conditioning tests, suggesting that TRPV1 regulates fear memory formation (Marsch et al., 2007). Under stressful conditions, *in vivo* activation of TRPV1 by intrahippocampal or intragastrical infusion of capsaicin prevented the impairing spatial memory retrieval caused by acute inescapable stress by placing mice on an elevated platform (Li et al., 2008), suggesting that activation of TRPV1 induces anti-stress effects.

*Trpa1* KO mice displayed decreased anxiety-like behaviors in elevated-plus maze, better performance in the fear conditioning and novel location recognition tests, and enhanced social recognition behavior (Lee et al., 2017) (Table 2), possibly due, at least in part, to the evidence that TRPA1 negatively regulates hippocampal functions potentially through the suppression of neurite outgrowth (Lee et al., 2017). However, there is a caveat regarding the interpretation of the behavioral data because *Trpa1* KO mice are suggested to exhibit impaired motor function through axonal bundle fragmentation, downregulation of myelin basic protein, and decreased mature oligodendrocyte population in the brain (Lee et al., 2017); therefore, the observed behavioral phenotypes could be merely due to impaired motor functions rather than hippocampal functions. Interestingly, TRPA1 in astrocytes is implicated to partially contribute to cuprizone-induced demyelination, which is frequently used for the animal model of multiple sclerosis (Kriszta et al., 2020), raising the possibility that there is a connection between TRPA1 expression or function and psychiatric disorders. Notably, another behavioral analysis using aged (18-month-old) *Trpa1* KO mice supports the notion that TRPA1 negatively regulates hippocampal functions in an age-dependent manner (Borbély et al., 2019) (Table 2). Thus, these lines of evidence suggest that TRPA1 is a negative regulator of brain functions, raising the possibility that there is a connection between TRPA1 expression or function and psychiatric disorders; however, further studies are required to validate this hypothesis.

## Perspective

**FIGURE 2:**
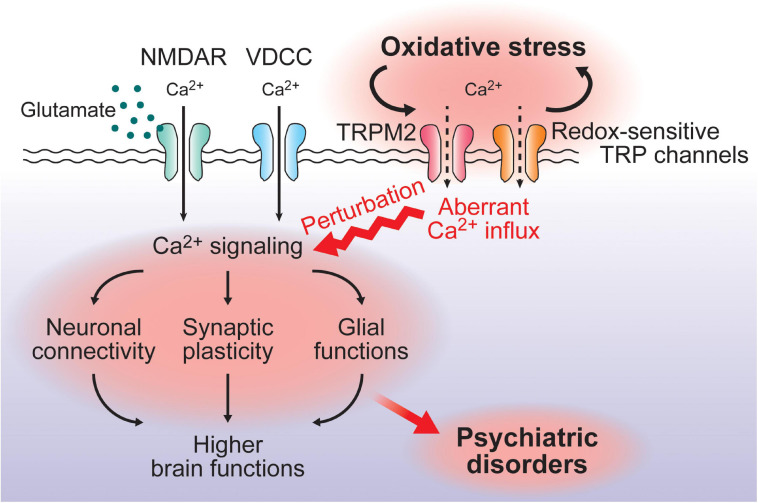
A putative model for the impact of redox-sensitive TRPchannels in the pathogenesis/pathophysiology of psychiatricdisorders. VDCC- or NMDAR-mediated Ca^2+^ signaling playsfundamental roles in neural functions such as neuronal connectivity, synaptic plasticity, and glial functions, contributing to higher brain functions. Oxidative stress alters expression and activities of redox-sensitive TRP channels, which further enhances oxidative stress and induces a perturbation of the Ca^2+^ signaling mediated by VDCC or NMDAR. This could be associated with the pathogenesis/pathophysiology of psychiatric disorders. Notably, SNPs of *TRPM2* have been identified as a risk factor for bipolar disorder.

As described above, accumulating evidence has indicated that most redox-sensitive TRP channels have significant impacts on higher brain functions even though the role of oxidative stress as the activation trigger of the channels in this context is not fully understood. Comprehensive analysis of gene expression in normal brains and the brains of patients with psychiatric disorders would delineate the significance of redox-sensitive TRP channels in these disorders, where notably, TRPM2 expression is enhanced in hippocampal tissue samples from patients with major depressive disorder (Ko et al., 2019), and, furthermore, TRPA1 is upregulated in astrocytes of the hippocampus in the mouse model of Alzheimer’s disease, in which oxidative stress is deeply involved (Lee et al., 2016). Given that oxidative stress is associated with psychiatric disorders and that the expression of redox-sensitive TRP channels is induced by oxidative stress partly via the activation of NRF2, an oxidant defense transcription factor (Takahashi et al., 2018), it is possible that upregulated redox-sensitive TRP channels are involved in the pathogenesis or pathophysiology of psychiatric disorders (Figures 1, 2).

The concept of excitation-inhibition balance in neuropsychiatricdisorders has been considered as a framework for investigating theirmechanisms (Sohal and Rubenstein, 2019). Given that TRPA1 has the highestoxidation sensitivity among the TRP channels (Takahashi et al., 2011b; Mori et al., 2016), it is likely that TRPA1 senses oxidative stressacutely and moderates the excitation-inhibition balance by regulating astrocyte resting [Ca^2+^]_*i*_, which affects inhibitory synapse efficacy through the reduction of GABA transport (Shigetomi et al., 2012). Recently, several studies have shown that treatment with novel TRPC4/TRPC5 channel inhibitors produce antidepressant and anxiolytic-like effects in mice, suggesting that TRPC4 and TRPC5 channels are novel molecular candidates for the treatment of psychiatric symptoms (Yang et al., 2015; Just et al., 2018) (Table 2). Thus, the investigation of redox-sensitive TRP channels in the pathogenesis/pathophysiology of psychiatric disorders may lead to the development of novel therapeutic strategies for the treatment of these disorders.

## Author Contributions

AN and NT generated the first draft of the manuscript. All authors contributed to the revisions.

## Conflict of Interest

The authors declare that the research was conducted in the absence of any commercial or financial relationships that could be construed as a potential conflict of interest.
